# Efficacy and Safety of Direct-Acting Antivirals in Kidney Transplantation From HCV-Viremic Donors to Negative Recipients: A Meta-Analysis

**DOI:** 10.3389/fmed.2022.802686

**Published:** 2022-05-18

**Authors:** Zepei Feng, Jinwei Zhang, Weilong Tan, Chunhui Wang, Qiong Chen, Chao Shen, Haozhi Fan, Yun Zhang, Peng Huang, Ming Yue

**Affiliations:** ^1^Department of Epidemiology and Biostatistics, Key Laboratory of Infectious Diseases, School of Public Health, Nanjing Medical University, Nanjing, China; ^2^Department of Anesthesiology, Affiliated Drum Tower Hospital of Medical College of Nanjing University, Nanjing, China; ^3^Institute of Epidemiology and Microbiology, Eastern Theater Command Centers for Disease Prevention and Control, Nanjing, China; ^4^Department of Information, First Affiliated Hospital of Nanjing Medical University, Nanjing, China; ^5^Department of Infectious Diseases, First Affiliated Hospital of Nanjing Medical University, Nanjing, China

**Keywords:** antiviral agents, HCV-viremia, Hepatitis C, kidney donors, kidney transplant, meta-analysis

## Abstract

**Background:**

With the development of direct-acting antiviral agents (DAAs), the research on kidney transplantation from Hepatitis C virus (HCV)-viremic donors to HCV-negative recipients has grown. The objective of this comprehensive analysis was to evaluate the efficacy and safety of DAAs in kidney transplantation from HCV-viremic donors to negative recipients.

**Methods:**

Multiple databases were searched for a systematic and comprehensive up to March 2022. The primary outcomes included the percentage of sustained virological response at week 12 after the end of treatment (SVR12), adverse events (AEs; any grade), and severe adverse events (SAEs) as the endpoints. Publication bias was examined by using the funnel plots and Egger's test.

**Results:**

In total, 16 studies with 454 subjects were included in the study and the pooled estimate of SVR12, AEs, and SAEs rates were 100.0% (95% CI: 99.2-100.0), 1.9%(95%CI: 0.0-4.9), and 0.0% (95%CI: 0.0-1.5). Subgroup analysis showed that pooled SVR12 rates were 100.0% (95%CI: 99.6-100.0) for genotype (GT)1a and 96.3% (95%CI: 83.3-100.0) for GT2; 100.0% (95%CI: 98.9-100.0) for DAAs treatments; and 100.0% (95%CI: 98.2-100.0) for prophylaxis subgroup. Egger's tests showed that no publication bias was found in this study.

**Conclusion:**

This comprehensive analysis showed the high efficacy and safety of DAAs in kidney transplantation from HCV-viremic donors to HCV-negative recipients.

**Systematic Review Registration:**

https://www.crd.york.ac.uk/PROSPERO/display_record.php?RecordID=246541.

## Introduction

Hepatitis C virus (HCV) infection affects around 180 million individuals worldwide, of which around 71 million people develop chronic HCV infections (1, 2). HCV may develop cirrhosis, hepatocellular carcinoma, and liver-related deaths (3), and 40% of the infected population may have extrahepatic manifestations due to HCV such as kidney injury, insulin resistance, and accelerated atherosclerosis (4). Increased HCV transmission on the heels of dramatic increases in opioid use among young adults.

A high rate of HCV infection was found in patients with chronic kidney disease (CKD). Kidney transplantation is the ultimate treatment for patients suffering from end-stage renal disease (ESRD) but is nevertheless limited by donor shortages (5). In the US, increased HCV transmission is occurring owing to the ongoing opioid epidemic, and opioid abuse and overdose have led to an increased supply of HCV-positive kidneys (6, 7). Meanwhile, the prevalence of ESRD has been rising, with around 95,000 candidates are on a waiting list to get a kidney transplant in America (8). According to the official data from the Organ Procurement and Transplantation Network (OPTN), the health of most candidates is gradually deteriorating. Some patients were more likely to die during the waiting time for a kidney, such as patients ≥ 60 years (9). The gap between the number of kidney transplant patients and the number of available organs will gradually enlarge. Actually, a kidney from HCV-positive donors has long remained an underutilized resource in America so far. Every year about 500 HCV-positive kidneys are discarded (10). The high-discard rate of HCV-positive kidneys was a matter of great concern because these kidneys are often younger and have fewer complications than common donors and with a lower kidney donor profile index (KDPI) (11–13). Such discard was largely driven by the previous clinical practice that HCV+ kidneys were provided only for HCV+ recipients, primarily because interferon (IFN)-based therapies were the main method of HCV, which were limited treatment options with poor efficacy and intolerable side effects (14, 15).

Compared with IFN-based therapies, direct-acting antiviral agents (DAAs) are extremely effective and well-tolerated in the general population with an advantageous side-effect profile, with virologic cure rates achieving 99% even among transplant recipients of the solid organs (16–18). The high efficacy and favorable safety of generic DAAs in real-world clinical practice have been evaluated by several international cohorts for all six major HCV genotypes (19, 20). DAAs broaden the range of patients on the transplant waiting list to allow the transplantation of organs from HCV-viremic donors into negative recipients. Various approaches should be taken into account to prevent the consequences of HCV in recipients of kidneys from HCV+ donors.

Some published literature has studied the safety and feasibility of transplanting organs from HCV-viremic donors. For the argument in the transplant community regarding the effects of using preemptive or post-transplantation treatment with DAAs, further research is needed to provide evidence. Our *post-hoc* analysis aims to evaluate the efficacy and safety of DAAs in kidney transplantation from HCV-viremic donors (D+) to negative recipients (R–).

## Method

### Search Strategy

Our protocol was performed following the Preferred Reporting Items for Systematic Review and Meta-analyses (PRISMA). This study was registered in the PROSPERO database (CRD42020133457). Two independent reviewers used multiple databases including PubMed, Embase, and Web of Science for a systematic and comprehensive search, which was last updated in March 2022 without language restrictions. The search strategy included (kidney transplant OR renal transplantation OR renal transplantations OR transplantations, renal OR transplantation, renal OR grafting, kidney OR kidney grafting OR transplantation, kidney OR kidney transplantations OR transplantations, kidney) AND (antiviral agents OR agents, antiviral OR antivirals OR antiviral drugs OR drugs, antiviral OR DAA OR direct acting antivirals OR direct acting antiviral) AND (Hepacivirus OR Hepaciviruses OR Hepatitis C-like viruses OR Hepatitis C-like Viruses OR Hepatitis C virus OR Hepatitis C viruses OR HCV). We also manually searched the reference cited in included articles and other relevant systematic reviews for additional appropriate studies to improve the search sensitivity.

### Selection Criteria

Studies were included in the qualitative analysis if they met all the following criteria: (1) recipients were HCV RNA negative at the time of transplant with no evidence of HCV infection by RT-PCR, (2) donors were HCV RNA positive, (3) SVR12 (sustained virological response 12 weeks after the end of treatment) could be measured, and (4) treatment with DAAs (including pre- and post-transplant DAAs therapy).

Studies were excluded if they met any of the following criteria: (1) recipients and/or donors were infected with HIV/HBV, (2) studies without SVR12 data, (3) studies without HCV RNA load, (4) case reports, conferences, meta-analyses, editorials, or reviews, and (5) cost-effectiveness, pharmacokinetics, or pharmacodynamics studies.

### Outcomes

The primary outcome was the percentage of sustained virological response at week 12 after the end of treatment (SVR12). SVR12 was defined as plasma HCV RNA < the lower limit of quantification at follow-up of 12 weeks after the end of treatment, which included post-transplant DAAs therapy (post kidney transplantation as positive HCV NAT tests) and pre-transplant DAAs therapy (before virus testing even during transplantation). The secondary outcomes, the percentages of HCV transmission from donors to recipients, were added for pre-transplant DAAs therapies. HCV transmission was defined as positive for HCV RNA in the post-transplant recipient. Stratified by the initiation time of DAAs therapy, HCV transmission rate was divided into the before renal transplant (RT) group and the after RT group for subgroup analysis. The incidence and intensity of adverse events (AEs) and serious adverse events (SAEs) were used to assess safety. Only studies reported the percentages of HCV transmission and AEs/SAEs were analyzed for the secondary outcomes and safety.

### Study Selection and Data Extraction

Study selection and data extraction were conducted independently by two researchers (ZPF and JWZ). Study selection was performed following the pre-designed inclusion and exclusion criteria. Studies were initially reviewed by titles and abstracts, and then potentially eligible studies were identified and screened again by full texts. The extracted data included the following: the name of the first authors, year, region, study type, publication type, single center or multi-center studies (setting), DAA regimens, the initiation time of DAAs therapy (drug node), duration of DAAs therapy (duration), sample size, the demographics of donors and recipients, waiting time for a kidney transplant (waiting list time), HCV genotype (GT), serum creatinine, glomerular filtration rate (GFR), study outcomes (SVR12, HCV transmission), and safety (AEs/SAEs). When the information or evidence was imprecise, or the opinions of two reviewers were not uniform, the full texts were accessed and discussions were made with a third reviewer.

### Quality Assessment

The quality of included non-randomized studies was assessed by the Methodological Index for Non-Randomized Studies (MINORS), which consists of eight methodological items for non-comparative studies. Each item was scored from 0 to 2, and 16 is the ideal score for non-comparative studies, indicating the highest study quality for non-randomized interventional studies, whereas 24 is the ideal score for comparative studies. The quality of observational studies was assessed using the Newcastle–Ottawa quality assessment scale (NOS) with a total score of nine. Low quality was scored as 0-5 points, moderate quality as 6-7 points, and high quality as 8-9 points. Two investigators independently assessed the quality of each included study.

### Statistical Analysis

Effect sizes were collected as pooled event incidences with corresponding 95% CI using the inverse variance method. In the case of 0 or 100% events, we estimated the incidence rate by using the Freeman–Tukey double arcsine transformation. The Cochran Q-statistics and *I*^2^ statistics were used to assess the heterogeneity across the included studies. The random-effect model (DerSimonian–Laird Method) was used in case of considerable heterogeneity, which was defined as *I*^2^ ≥ 50%; the fixed effects model (Inverse–Variance Method) was used when *I*^2^ <50%. Subgroup analysis was used to understand the potential sources of heterogeneity. Publication bias was examined by using the funnel plot and Egger's test. If there is a publication bias, the Duval and Tweedie nonparametric trim and fill analysis will be performed to account for publication bias. The sensitivity analysis was conducted to explore the effect of each study on effect sizes. All the statistical tests were two-sided, with a *P*-value < 0.05 considered to be statistically significant. All the statistical analyses were performed using the R version 4.0.4.

## Results

### Study Selection and Basic Information

**Figure 1:**
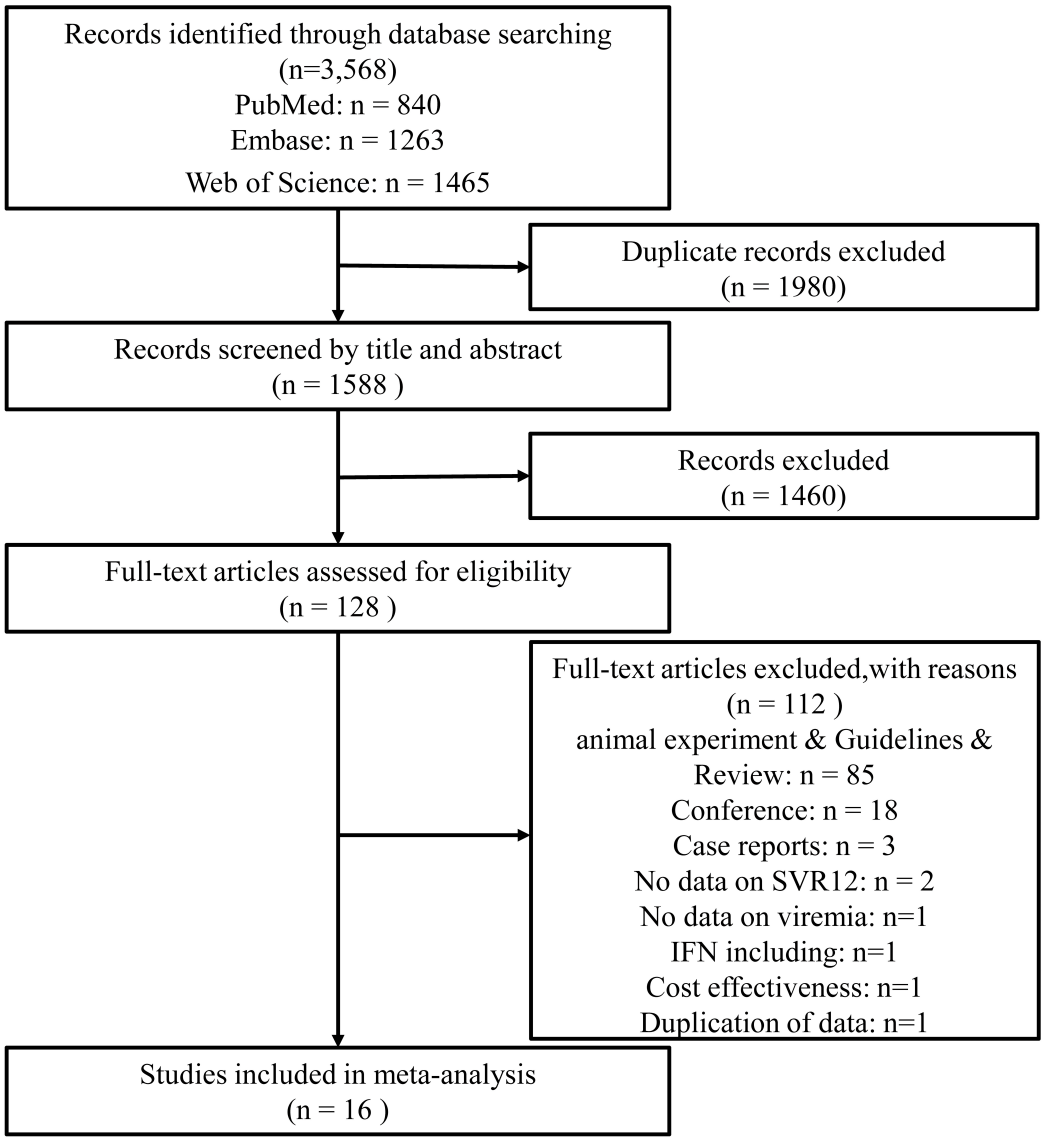
The flow diagram of literature screening and following the preferred reporting items of systematic reviews and meta-analyses (PRISMA).

The initial search identified 3,568 eligible studies. After deleting 1,980 duplicate studies, the titles and abstracts of 1,460 articles were screened. A total of 124 articles were selected for full-text reading and 112 articles were excluded for different reasons. Eventually, 16 articles (21–36) were included in this study (Figure 1). The 16 studies were published during 2018–2022, including 15 full articles and 1 letter, which were conducted in 3 regions: the USA (14 articles), Germany (1 article), and China (1 article). In total, 9 studies conduct DAAs therapies after kidney transplantation when the recipients detected positive HCV RNA; 7 studies started DAAs prophylaxis therapies before or during the transplantation. The total number of study recipients was 454 and the donors were 394. The characteristics of these 16 studies were described in Tables 1, 2.

**Table 1:** Characteristics of the studies included in this comprehensive analysis.

**References**	**Study design**	**Publication type**	**Region**	**Setting**	**Protocol**	**Drug node**	**Sample size**		**Duration (weeks)**
							**Donors**	**Recipients**	
Gupta et al. (21)	Clinical trial	Full	USA	Single center	pre-transplant therapy	Before RT	49	50	12w
Kapila eta l. (22)	Clinical trial	Full	USA	Single center	post-transplant therapy	\	41	41	12w
Sise et al. (23)	Clinical trial	Full	USA	Single center	pre-transplant therapy	After RT	6	8	12w
Molnar et al. (26)	Clinical trial	Full	USA	Single center	post-transplant therapy	\	65	65	12w
Reese et al. (24)	Clinical trial	Full	USA	Single center	post-transplant therapy	\	15	20	12w
Jandovitz et al. (25)	Clinical trial	Full	USA	Single center	post-transplant therapy	\	25	25	12w
Durand et al. (27)	Clinical trial	Full	USA	Single center	pre-transplant therapy	After RT	10	10	12/16w
Friebus et al. (28)	Clinical trial	Full	Germany	Single center	post-transplant therapy	\	5	7	12w
Graham et al. (29)	Clinical trial	Full	USA	Single center	post-tran splant therapy	\	30	30	12.86w
Sise et al. (30)	Clinical trial	Full	USA	Multicenter	pre-transplant therapy	After RT	30	30	8w
Terrault et al. (31)	Clinical trial	Full	USA	Multicenter	post-transplant therapy	\	10	10	12w
Durand et al. (32)	Clinical trial	Letter	USA	Single center	pre-transplant therapy	After RT	10	10	4w
Chen et al. (33)	Retrospective study,	Full	China	Single center	pre-transplant therapy	Before RT	15	26	12w
Hudson et al. (34)	Retrospective Chart review	Full	USA	Single center	post-transplant therapy	\	NA	22	12w
Gupta et al. (35)	Clinical trial	Full	USA	Single center	pre-transplant therapy	Before RT	50	50	12w
Concepcion et al. (36)	Cohort study	Full	USA	Single center	post-transplant therapy	\	32	50	12w

*Setting, single center or multi-center studies; drug node, the initiation time of DAAs therapy; RT, renal transplant*.

**Table 2:** Patient characteristics of the studies enrolled in this comprehensive analysis.

**References**	**Recipients**				**Donors**			**HCV genotype**	**DAA regimens**	**DAAINT (Days)**	**RASs in NS3 or NS5A**
	**Age**	**Gender (M)**	**Race (African American)**	**ESRD cause (CKD,%)**	**KDPI(%)**	**KDPI sans HCV(%)**	**HCV RNA (IU/ml)**				
Gupta et al. (21)	60(36-76)	32	62%	NA	62 ± 18	37 ± 18	2.65E + 06(2.56E + 02 – 7.4E + 06)	GT1,2,3	GLE/PIB,SOF/VEL ± RBV,EBR/GZR ± RBV	before RT	3
Kapila eta l. (22)	69.5(32-81)	20	17%	NA	54(25-99)	NA	NA	GT1,2,3,4	LDV/SOF ± RBV,GLE/PIB,SOF/VEL ± RBV	72(7-198)	1
Sise et al. (23)	55.9(9.4)	6	0%	12.5	31(29-65)	NA	NA	GT1	EBR/GZR ± RBV	before RT	0
Molnar et al. (26)	52 ± 11	40	82%	6	50 ± 16	NA	NA	GT1,2,3	LDV/SOF ± RBV,GLE/PIB,SOF/VEL ± RBV	76(68,88)	NA
Reese et al. (24)	56.3(6.7)	14	NA	15	46(33,54)	NA	2.91E + 05(1.33E + 02 – 2.05E + 07)	GT1	EBR + GZR ± R	3	3
Jandovitz et al. (25)	57.7 ± 10.4	19	NA	NA	49(38-66)	NA	NA	GT1,3	LDV/SOF ± RBV,SOF/VEL ± RBV	13(8-22)	0
Durand et al. (27)	71(65-72)	8	NA	30	45(32, 37)	NA	6.24E + 04 (< LLOQ – 2.09E + 06)	GT1,2,3	EBR/GZR,EBR/GZR + SOF	before RT	0
Friebus et al. (28)	NA	4	NA	14	NA	NA	< LLOQ	GT1,3	LDV/SOF ± RBV,SOF/VEL ± RBV	7(5-37)	1
Graham et al. (29)	69.4 ± 4.6	19	NA	NA	62.8 ± 17.1	39.5 ± 19.3	4.53E + 06(1.72E + 04 – 1.72E + 07)	GT1,2,3,4	GLE/PIB,SOF/VEL ± RBV	9(5-41)	NA
Sise et al. (30)	57(51-60)	21	30%	10	NA	NA	NA	GT1,2,4	GLE/PIB	(2-5)	NA
Terrault et al. (31)	54(52,57)	5	27%	NA	52 (40.5, 61.5)	NA	NA	NA	SOF/VEL ± RBV	16.5(9.8,24.5)	NA
Durand et al. (32)	67(40-75)	7	20%	20	60(29-76)	NA	NA	GT1,3	GLE/PIB	NA	NA
Chen et al. (33)	42(20,73)	19	0%	12	NA	NA	(5.83 – 1.1E + 06)	GT1,2,3	SOF/VEL ± RBV	before RT	NA
Hudson et al. (34)	62 ± 11	14	27%	14	NA	NA	NA	GT1,3	GLE/PIB,SOF/VEL ± RBV	41 ± 13	NA
Gupta et al. (35)	54 ± 13	26	66%	NA	69 ± 24	50 ± 29	NA	GT1	GLE/PIB,SOF/VEL ± RBV	before RT	NA
Concepcion et al. (36)	56	34	48%	0%	NA	NA	NA	GT1,2,3	LDV/SOF ± RBV, GLE/PIB,SOF/VEL ± RBV	29 ± 11	NA

*M, male; CKD, cystic kidney disease; DAAINT, Time from transplant to initiation of DAA, days; NA, not applicable; GLE/PIB, glecaprevir/pibrentasvir; SOF/VEL ± RBV, sofosbuvir/velpatasvir ± ribavirin; EBR/GZR ± RBV, elbasvir/grazoprevir ± ribavirin; LDV/SOF ± RBV, ledipasvir/sofosbuvir ± ribavirin*.

### Quality of the Included Studies

Supplementary Tables S1, S2 showed the quality assessment scores. In total, 13 non-randomized studies were assessed by MINORS. Scores between 0 and 4 correspond to a high risk of bias, scores between 5 and 10 correspond to a moderate risk of bias, and scores between 11 and 16 correspond to a low risk of bias (38). Among them, a median MINORS score was 12 (range: 11–17), which showed a low risk of bias. In three observational studies assessed by using NOS, one was of high quality, and two were of low quality.

### Efficacy of Outcomes

#### SVR12

**Figure 2:**
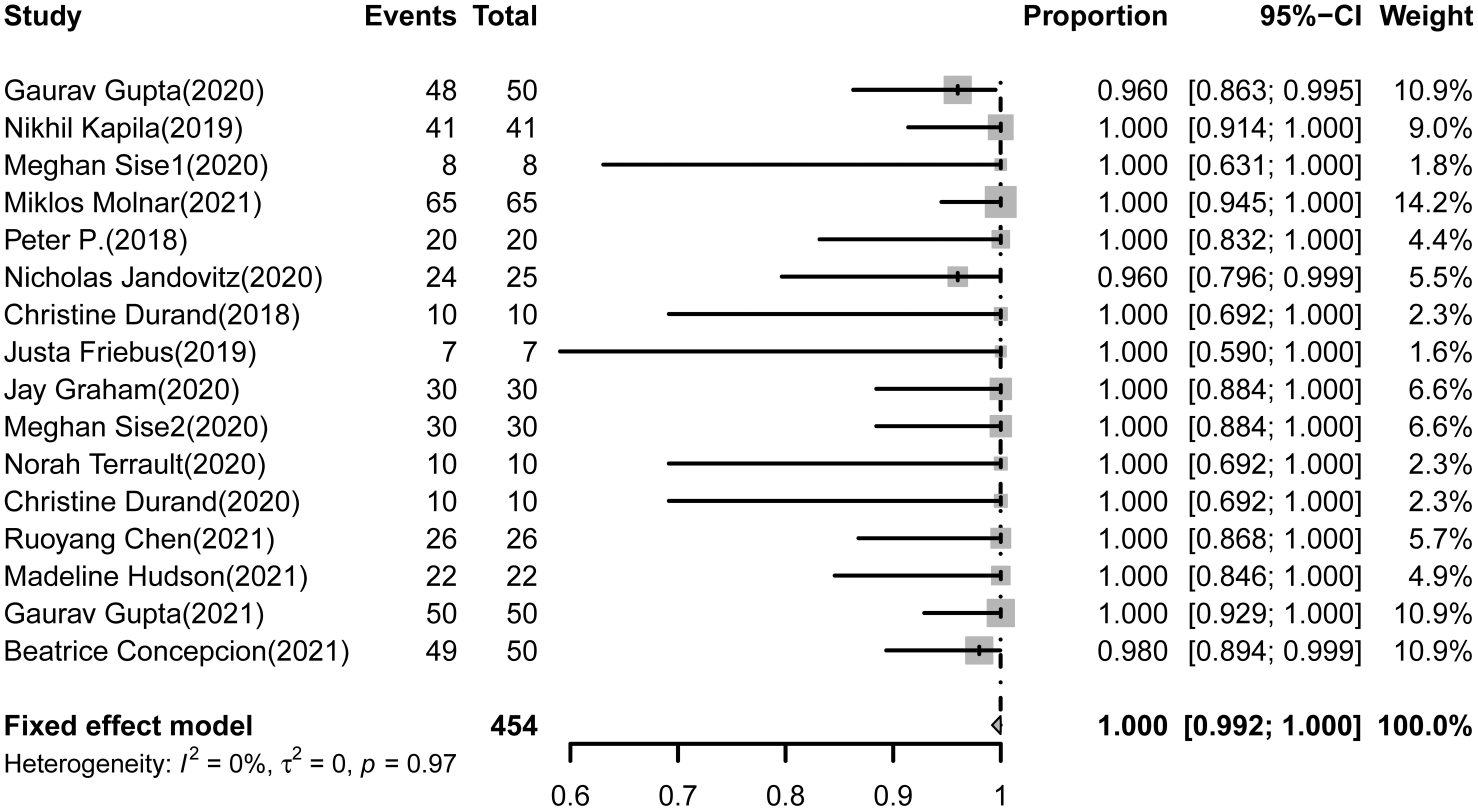
Forest plot of pooled SVR rates of DAAs treatment for HCV-negative recipients received HCV+ kidneys.

All 16 studies (454 cases) reporting SVR12 rates of DAAs in HCV-negative recipients received HCV+ kidneys. The pooled estimations of the SVR12 rate from the fixed-effect model was 100.0% (95%CI: 99.2-100.0, *I*^2^= 0.0%, *P* = 0.97) (Figure 2).

#### HCV Transmission

In total, seven DAAs prophylaxis studies reported the rate of HCV transmission rates from donors to recipients. Among 184 recipients included in the analysis, the HCV transmission rate was 33.1% (95%CI: 7.8-64.4) and a substantial level of heterogeneity was observed (*I*^2^= 94.0%, *P* < 0.01) (**Table 6**).

### Subgroup Analysis

#### Subgroup Analysis of the Overall SVR12 Rate

Based on settings, protocols, GTs, regimens and durations, subgroup analysis was conducted as detailed in Table 3. The rate of SVR 12 was 100.0% (95%CI: 95.9-100.0) in multi-center studies and 100.0% (95%CI: 99.0-100.00) in single-center studies. Nearly, 100.0% of patients in both groups achieved SVR12, 95%CI: 98.9-100.0 for post-transplant subgroup, and 95%CI: 98.2-100.0 for pre-transplant subgroup. In total, 14 studies that included 289 patients recorded GTs data for subgroup analysis. In GTs subgroups, the SVR12 rates in GT1, GT1a, GT1b, GT2, GT3, GT1a/3, and GT4 were 100.0% (95%CI: 77.2-100.0), 100.0% (95%CI: 99.6-100.0), 100.0% (95%CI: 81.1-100.0), 96.3% (95%CI:83.3-100.0), 100.0% (95%CI: 98.9-100.0), 100.0% (95%CI: 21.3-100.0), and 100.0% (95%CI: 21.3-100.0), respectively. By different DAAs regimens, the patients' SVR12 rate was 100.0% (95%CI: 100.0-100.0) in glecaprevir/pibrentasvir (GLE/PIB) subgroup, was 100.0% (95%CI: 100.0-100.0) in sofosbuvir/velpatasvir ± ribavirin (SOF/VEL ± RBV) subgroup, was 100.0% (95%CI: 94.2-100.0) in elbasvir/grazoprevir ± ribavirin (EBR/GZR ± RBV) subgroup, and was 99.0% (95%CI: 86.5-100.0) in ledipasvir/sofosbuvir ± ribavirin (LDV/SOF ± RBV) subgroup.

**Table 3:** SVR12 by settings, genotypes, regimens, durations, and protocols.

**Response**	**SVR12**		**Heterogeneity**		P^**^-value	**Studies**
	**Total, *n/N***	**Rate(95%CI)**	***I* ^2^**	*P* ^*^		***N***
Overall	450/454	100.0(99.2-100.0)	0.0	0.97		16
By settings					0.82	
Single-center	410/414	100.0(99.0-100.0)	0.0	0.94		14
Multi-center	40/40	100.0(95.9-100.0)	0.0	0.73		2
By genotypes					0.72	
1	8/8	100.0(77.2-100.0)	0.0	0.88		2
1a	186/189	100.0(99.6-100.0)	0.0	0.96		13
1b	10/10	100.0(81.1-100.0)	0.0	0.99		5
1a/3	2/2	100.0(21.3-100.0)	0.0	1.00		2
2	16/17	96.3(83.3-100.0)	0.0	0.84		7
3	61/61	100.0(98.9-100.0)	0.0	1.00		10
4	2/2	100.0(21.3-100.0)	0.0	1.00		2
By regimens					0.11	
VEL + SOF ± R	167/169	100.0(100.0-100.0)	0.0	0.78		10
GLE + PIB	176/176	100.0(100.0-100.0)	0.0	0.99		8
EBR + GZR ± R	37/38	100.0(94.2-100.0	16.0	0.31		4
LDV + SOF ± R	30/32	99.0(86.5-100.0)	0.0	0.98		4
EBR/GZR ± SOF	3/3	\	\	\		1
By duration					0.66	
≥12w	380/384	100.0(99.0-100.0)	0.0	0.91		13
<12w	70/70	100.0(97.6-100.0)	0.0	0.93		3
By protocols					0.76	
pre-transplant therapy	182/184	100.0(98.2-100.0)	0.0	0.83		7
post-transplant therapy	268/270	100.0(98.9-100.0)	0.0	0.90		9

* *Test of heterogeneity*.

** *Test for subgroup differences*.

#### Subgroup Analysis of Protocol

For pre-transplant DAAs therapy group and post-transplant DAAs therapy group, we conducted subgroup analysis by different GTs and DAAs. In DAAs prophylaxis studies, the SVR12 rates in GT1a, GT1b, GT2, and GT3, were 100.0% (95%CI: 96.3-100.0), 100.0% (95%CI: 21.3-100.0), 95.0% (95%CI: 51.2-100.0), and 100.0% (95%CI: 91.3-100.0), respectively. By different DAAs regimens, the patients' SVR12 rates using GLE/PIB, SOF/VEL ± RBV, and EBR/GZR ± RBV were 100.0% (95%CI: 100.0-100.0), 99.6% (97.1-100.0), and 99.0% (95%CI: 83.4-100.0), respectively (Table 4).

**Table 4:** Subgroup analysis of SVR12 for pre-transplant.

**Response**	**SVR12**		**Heterogeneity**		P^**^-value	**Studies**
	**Total, *n/N***	**Rate(95%CI)**	***I* ^2^**	*P* ^*^		***N***
Pre-transplant	182/184	100.0(98.2-100.0)	0.0.	0.83		7
By genotypes					0.89	
1a	49/50	100.0(96.3-100.0)	0.0	0.97		6
1b	2/2	100.0(21.3-100.0)	0.0	1.00		2
1a/3	1/1	\	\	\		1
2	6/7	95.0(51.2-100.0)	0.0	0.65		4
3	16/16	100.0(91.3-100.0)	0.0	0.96		4
4	1/1	\	\	\		1
By regimens					0.49	
GLE + PIB	43/43	100.0(100.0-100.0)	0.0	0.81		4
VEL + SOF ± R	125/126	99.6(97.1-100.0)	0.0	0.59		3
EBR/GZR ± SOF	16/17	99.0(83.4-100.0)	24.0	0.27		3

* *Test of heterogeneity*.

** *Test for subgroup differences*.

In post-transplant studies, the SVR12 rates in GT1a, GT1b, GT2, and GT3, were 100.0% (95%CI: 98.5-100.0), 100.0% (95%CI: 77.5-100.0), 100.0% (95%CI: 83.9-100.0), and 100.0% (95%CI: 96.9-100.0), respectively. By different DAAs regimens, the patients' SVR12 rates using LDV/SOF ± RBV, SOF/VEL ± RBV, and GLE/PIB were 99.0% (95%CI: 86.5-100.0), 100.0% (95%CI: 97.1-100.0), and 100.0% (95%CI: 98.7-100.0), respectively (Table 5).

**Table 5:** Subgroup analysis of SVR12 for post-transplant.

**Response**	**SVR12**		**Heterogeneity**		P^**^-value	**Studies**
	**Total, *n/N***	**Rate(95%CI)**	***I* ^2^**	*P* ^*^		***N***
Post-transplant	268/270	100.0(98.9-100.0)	0.0	0.90		9
By genotypes					0.93	
1a	137/139	100.0(98.5-100.0)	0.0	0.74		7
1b	8/8	100.0(77.5-100.0)	0.0	0.94		3
1a/3	1/1	\	\	\		1
2	10/10	100.0(83.9-100.0)	0.0	0.90		3
3	45/45	100.0(96.9-100.0)	0.0	0.99		6
4	1/1	\	\	\		1
By regimens					0.04	
LDV + SOF ± R	30/32	99.0(86.5-100.0)	0.0	0.98		4
GLE + PIB	133/133	100.0(98.7-100.0)	0.0	0.98		4
VEL + SOF ± R	41/41	100.0(97.1-100.0)	0.0	1.00		7

* *Test of heterogeneity*.

** *Test for subgroup differences*.

#### Subgroup Analysis of HCV Transmission

Stratified by drug nodes, the HCV transmission rate was 4.5% (95%CI: 0.1-13.1) in the before RT subgroup and was 68.4% (95%CI: 50.0-84.5) in the after RT subgroup. The subgroup analysis slightly reduced the heterogeneity (before RT: *I*^2^= 62.0%; after RT: *I*^2^= 39.0%, respectively), and a significant difference was observed between the two subgroups (*P* < 0.01) (Table 6).

**Table 6:** Transmission by drug node.

**Response**	**Transmission**		**Heterogenelty**		P^**^-value	**Studies**
	**Total, *n/N***	**Rate(95%CI)**	***I^**2**^***	*P* ^*^		***N***
overall	48/184	33.1(7.8-64.4)	94	<0.01		7
By drug node					<0.01	
Before RT	8/126	4.5(0.1-13.1)	62	0.07		3
After RT	43/58	68.4(50.0-84.5)	39	0.18		4

* *Test of heterogeneity*.

** *Test for subgroup differences*.

### Safety

In total, nine studies reported AEs, and ten studies reported SAEs related to DAAs therapy. The pooled rates of AEs and SAEs were 1.9% (95%CI: 0.0–4.9) and 0.0% (95%CI: 0.0–1.5), respectively. The main SAEs were fibrosing cholestatic hepatitis recorded in two studies.

### Publication Bias and Sensitivity Analysis

The funnel plot for the pooled estimations of the SVR12 rate was asymmetrical (Supplementary Figure S2). The results of Egger's test showed no publication bias in this study (*t* = −0.92, *P* = 0.37) (Supplementary Figure S1). Furthermore, the sensitivity analysis of the SVR12 rate revealed that all effect sizes did not depend on a single study (Supplementary Figure S3).

## Discussion

This meta-analysis of the clinical trials aims to evaluate the efficacy and safety of direct-acting antivirals in kidney transplantation from HCV-viremic donors to HCV-negative recipients. These results indicated that DAAs therapy for patients with kidney transplantations of HCV NAT+ donors can achieve high SVR12 rates, regardless of sex, age, settings, protocols, GTs, regimens, and durations. There are a few numbers SAEs related to the HCV protocol. In summary, kidneys from HCV NAT+ donors can be safely transplanted into HCV-negative recipients following DAA therapy, which is an effective and secure retreatment option to expand the donor pool and decrease organ discard.

In this study, the pooled SVR12 was100.0% (95%CI: 99.2-100.0), which showed that DAA regimens were highly effective. The results were similar to a recent study by Yang et al. (39). In terms of different DAA regimens, no significant differences were found in subgroups, which is also similar to several published studies of DAA regimens. All the DAA mentioned in the studies are recommended regimens by the American Association for the study of liver diseases (AASLDs) (40). For GTs, there is not only a high SVR12 rate but also no difference in a subgroup analysis of DAA regimens. The SVR12 rate of the GT3 subgroup is also high (100.0%), although HCV GT3-infected patients have been traditionally described as difficult-to-treat than other GTs (41). The development of DAAs allows it possible to use all GTs of the donor's kidneys instead of discarding them, the highly SVR12 rate can provide evidence for the use of HCV+ kidneys.

A low rate of HCV transmission was found in this meta-analysis, with significant heterogeneity (*P* < 0.01). On the one hand, the small sample size limited the result and the gap between the patient number in each study was large. Therefore, more evidence is needed to confirm. A clinical study reported a heart and lung transplantation from HCV-viremia donors, in which HCV-negative recipients received SOF/VEL therapy from HCV-viremia donors, beginning within a few hours after transplantation. In the study, a total of 42 of 44 recipients (95%) had a detectable Hepatitis C viral load immediately after transplantation (42). Therefore, we inferred that the timing to begin DAAs therapy will also affect HCV spread. On the other hand, in the subgroup analysis by drug node, heterogeneity was slightly reduced (before RT: *I*^2^= 62.0%; after RT: *I*^2^= 39.0%, respectively), and a significant difference was observed between two subgroups (*P* < 0.01). In addition, the detectable viremia of recipients after kidney transplantations in all studies was transience and the viral loads were steadily decreased, which was suggestive of the presence of residual viral RNA from donor kidney, rather than active infection and replication, consistently with the previous study that viral loads of donors were significantly associated with viremia in the organ recipients. It is possible to administer DAAs prior to transplant to block the viral transmission and replication in the kidney recipients. Although the transmission rate is not very low, DAAs can further reduce the later infection rate and the viral loads after transplantation. Considering the cost-effectiveness, insurance coverage for DAA drugs might frequently be denied due to a presumed lack of “medical necessity” and the high costs of DAAs have led public and private insurers to restrict access to these medications (43, 44). Thus, a prophylaxis therapy that mitigates the need for a full course of DAA therapy might make this method more appealing to the patients and providers.

However, the initiation time of DAAs therapy in HCV-negative recipients is controversial (45, 46). Our study results indicate that there are no significant differences between the pre-transplant DAAs therapy group and the post-transplant DAAs therapy group. Both groups get a high SVR12 rate. In the process of organ transplantation, it is feasible to suffer from viral active infection and replication due to the intense immunosuppression (47). The immunological risks include acute hepatitis, fibrosing cholestatic hepatitis, acute/chronic rejection, etc. The initiation of DAAs regimens administered very early would likely mitigate some of these risks. On the other hand, there are novel combinations of DAAs with pan-genotypic activity by the development of DAAs, which were licensed even for patients with eGFR <30 ml/min/1.73 m^2^. In November 2019, the U.S. Food and Drug Administration (FDA) amended the package inserts for sofosbuvir-containing regimens to allow use in patients with renal disease, including those with an eGFR ≤ 30 ml/min (40). It is possible to use DAAs therapy during the perioperative period with low-kidney function. For the pharmacokinetics of DAAs, a study reported that sofosbuvir's AUCs were higher in the subjects with mild, moderate, and severe renal impairment in patients with renal function impairment, whereas its active metabolite, GS-331007 AUCs were also higher (48). Furthermore, sofosbuvir and GS-331007 AUC were 28 and 1,280% higher when sofosbuvir was dosed 1 h prior to hemodialysis compared with 60 and 2,070% higher when sofosbuvir was dosed 1 h after hemodialysis, respectively (37). Comparing ESRD and healthy participants, geometric mean ratios (GMRs) for EBR and GZR AUC were 0.99 (0.75-1.30) and 0.83 (0.56-1.22) on hemodialysis (HD) days (49). The aforementioned reasons provide some theoretical basis for the implementation of the prophylaxis protocol. Although the sample size of prophylaxis was small (*n* = 7), which caused poor accuracy and reliability so more prophylaxis cases should be included to obtain enough evidence, the results of our research also support the scientific hypothesis, which can be used as reference evidence for the treatment plan in the future.

Among all the included studies, patients who failed to achieve SVR12 were reported in only three studies. Two patients with treatment failure in the first study were found to have resistance-associated substitutions (RASs) in the NS3/4A or NS5A region, not at the time of baseline (21). RASs were produced by the error-prone replication of HCV that could decrease the efficacy of the DAAs regimens (50). In the second study, a very high viral load was detected in one recipient at SVR12, with a mixed HCV 1a and 2b subtypes infection (25). This patient received an HCV+ kidney with 2b subtype, but was treated with LDV/SOF based for the HCV 1a genotype, which did not respond to 2b subtype. In the third study, one HCV 1a-infected recipient, treated with LDV/SOF and without viral resistance testing, neither achieved SVR-4 nor SVR12 with unknown cause, but eventually obtained SVR12 after retreating with sofosbuvir/velpatasvir/voxilaprevir (36). From the aforementioned studies, it is essential to accurately capture the HCV genotyping and RASs information of donors and recipients to make sure that patients can benefit from the best treatment for their condition.

According to the studies that reported renal function at 6 months or 12 months post-transplant, all the patients had normal levels of the glomerular filtration rate or creatinine, indicating that the recent outcomes of kidney transplantation were relatively good. The observations were consistent with a recent study by Potluri et al., who showed no significant difference in the outcomes (12-month eGFR post-transplant) between HCV+ kidneys recipients and HCV- kidneys, respectively (51).

There are several limitations to this study. First, for the ethical and medical considerations, most of these included medication studies were not designed as randomized controlled trials, and thus, making the relative risk for the various subgroups could not be evaluated. Second, the most included studies were from America, potentially limiting the choice of DAAs regimens and our results' applicability to the rest of the world. Third, subgroup analyses were not conducted because of inadequate data on kidneys recipients.

Nevertheless, our comprehensive analysis exhibited several strengths. First, this meta-analysis was the first to evaluate the efficacy and safety of DAAs in kidney transplantation from HCV-viremic donors (D+) to negative recipients (R–). We screened 12 studies including 306 individuals, which allowed us to accurately assess the pooled SVR12 rates, viral transmission, and SAEs rates of populations who received HCV-positive kidneys. Given the low heterogeneity shown in the most included studies, we are confident that the results in this study are reliable and can provide a reference for clinicians.

## Conclusion

This comprehensive analysis showed the high efficacy and safety of DAAs in kidney transplantation from HCV-positive donors to HCV-negative recipients. DAAs therapy should be given early to reduce the risk of HCV infection post-transplant. The findings of this study may help to expand the donor pool and shorten the waiting time for kidney transplantation.

## Data Availability Statement

The original contributions presented in the study are included in the article/Supplementary Material, further inquiries can be directed to the corresponding author/s.

## Author Contributions

ZPF, PH, MY, and JWZ participated in the design of the study. ZPF, JWZ, CHW, YZ, and CS took charge of literature retrieval, data collection and quality control. ZPF, WT, and HZF performed the statistical analysis. QC, YZ, and MY contributed to analysis. ZPF, JWZ, and PH wrote the paper. All authors read and approved the final manuscript.

## Funding

This study was supported by the National Natural Science Foundation of China (81773499), Key Project of Natural Science Foundation of Yunnan Province (2019FA005), Science Foundation for Distinguished Young Scholars of Jiangsu Province (BK20190106), and Priority Academic Program Development of Jiangsu Higher Education Institutions (PAPD).

## Conflict of Interest

The authors declare that the research was conducted in the absence of any commercial or financial relationships that could be construed as a potential conflict of interest.

## Publisher's Note

All claims expressed in this article are solely those of the authors and do not necessarily represent those of their affiliated organizations, or those of the publisher, the editors and the reviewers. Any product that may be evaluated in this article, or claim that may be made by its manufacturer, is not guaranteed or endorsed by the publisher.
